# Primary and submovement control of aiming in C6 tetraplegics following posterior deltoid transfer

**DOI:** 10.1186/1743-0003-11-112

**Published:** 2014-07-23

**Authors:** Mark A Robinson, Digby Elliott, Spencer J Hayes, Gabor J Barton, Simon J Bennett

**Affiliations:** 1 Research Institute for Sport and Exercise Sciences, Liverpool John Moores University, Tom Reilly Building, Byrom Street, Liverpool L3 3AF, UK; 2 Department of Kinesiology, McMaster University, Ontario, Canada

**Keywords:** Tetraplegia, Aiming, Submovement, Upper-limb control, Muscle transfer

## Abstract

**Background:**

Upper limb motor control in fast, goal-directed aiming is altered in tetraplegics following posterior-deltoid musculotendinous transfer. Specifically, movements have similar end-point accuracy but longer duration and lower peak velocity than those of age-matched, neurotypical controls. Here, we examine in detail the interplay between primary movement and submovement phases in five C6 tetraplegic and five control participants.

**Methods:**

Aiming movements were performed in two directions (20 cm away or toward), with or without vision. Trials that contained a submovement phase (i.e., discontinuity in velocity, acceleration or jerk) were identified. Discrete kinematic variables were then extracted on the primary and submovements phases.

**Results:**

The presence of submovements did not differ between the tetraplegic (68%) and control (57%) groups, and almost all submovements resulted from acceleration and jerk discontinuities. Tetraplegics tended to make a smaller amplitude primary movement, which had lower peak velocity and greater spatial variability at peak velocity. This was followed by a larger amplitude and longer duration secondary submovement. Peak velocity of primary movement was not related to submovement incidence. Together, the primary and submovement phases of both groups were equally effective in reducing end-point error.

**Conclusions:**

C6 tetraplegic participants exhibit some subtle differences in measures of motor behaviour compared to control participants, but importantly feedforward and feedback processes work effectively in combination to achieve accurate goal-directed aiming.

## Background

Tetraplegics with a spinal cord lesion at C6 typically have normal use of the biceps brachii and wrist extensor muscles but paralysis of the triceps brachii, wrist flexors and all finger and thumb muscles. The functional imbalance at the elbow between the biceps brachii and triceps brachii means that there is no antagonist to elbow flexion or agonist for elbow extension. This has consequences for elbow flexion movements, with tetraplegics exhibiting greater movement amplitudes, as well as longer acceleration and deceleration times than unimpaired controls [1]. Other studies, however, have shown that the aiming movements of tetraplegics retain many kinematic similarities to the movement of neurotypical control participants [2,3].

A solution to permanently replace the function of the paralysed triceps brachii is posterior-deltoid (PD) musculotendinous transfer [4]. Recent evaluation of the effects of PD transfer on aiming movements with the upper limb showed that typical spatial and temporal characteristics are not completely restored compared to age-matched, neurotypical control participants [5]. Specifically, although tetraplegics who had undergone PD transfer took no more time than controls to plan and initiate aiming movements, and were no more reliant on vision for online regulation, they maintained overall accuracy by reducing movement velocity and lengthening movement time. It was suggested that tetraplegic participants had learned to adopt a feedforward and feedback control that minimizes the spatial variability associated with a noisier neuromuscular system; for a discussion of strategic influences on motor control see [6,7]. In other words, tetraplegics exhibited longer movement time because they recognized moving at a faster speed similar to neurotypical control participants would produce more variable trajectories [8,9], which would then require online correction. Additional support for this interpretation was evident in the relatively coarse classification (i.e., zero crossing in velocity or acceleration profile) and analysis of submovements (i.e., total number), which revealed no group differences.

The aim of the current study was to describe in more detail the primary movement and submovement phases of discrete adapted aiming movements performed by tetraplegics with PD transfer. Extending upon previous work where all trials were considered [5], here we reanalysed only those trials containing a primary and submovement phase (>50%). In addition to measures of movement kinematics, we quantified the frequency and distribution (velocity, acceleration or jerk discontinuity) of submovements, as well as their amplitude and duration. We also sought to determine if peak velocity is related to the incidence of submovements on a trial-by-trial basis, and finally if submovements reduced end-point error [10]. Aiming movements were performed with a ball transfer unit that enabled participants to perform a ‘frictionless’ multi-directional aiming movement away from or toward the body. Accordingly, we were able to examine if performing aiming movements that required a different coordinated recruitment of the agonist and antagonist muscles impacted the control of primary movement and submovement phases. In addition, the discrete aiming task was performed while wearing liquid crystal goggles such that on half the trials participants were prevented from seeing either their hand or the target during the execution of the aiming movement. In this way, we were able to investigate the influence of having access to visual and/or proprioceptive feedback. Finally, by comparing these data to those exhibited by age-matched, neurotypical controls, it was our intention to provide further insight into the motor control strategies of tetraplegics with PD transfer.

## Materials and methods

### Participants

Five male C6 tetraplegics (mean age 39 ± 6 years) and five male aged-matched unimpaired controls (mean age 38 ± 7 years) participated in this study (for details see Table 1). All provided informed consent and were free to withdraw at any time. All tetraplegics had undergone transfer of the PD muscle onto the triceps brachii as fully described previously [5], which was performed by the same surgeon. Following surgery and appropriate rehabilitation, all tetraplegic participants could actively extend the elbow against gravity which is indicative of a motor score of 3–5 for elbow extension post-surgery. All tetraplegic participants were capable of reaching > 20 cm in the horizontal plane as required in this experiment, but had impaired digit control and consequently could not point with the index finger. Four out of five participants were using a manual wheelchair for independent daily transport.

**Table 1:** Details of injury classification and surgical history for the tetraplegic participants

			**Level of injury**			**Time elapsed since**			
**Participant**	**Age (yrs)**	**Sex**	**Skeletal**	**Neurological**	**Tested arm**	**Injury (yrs)**	**Operation (yrs)**	**Uni/bilateral transfer**	**ASIA**
1	36	Male	C5/6	C6	R	21	14	Unilateral	C
2	44	Male	C5	C6	L	23	13	Bilateral	B
3	36	Male	C5	C6	R	16	9	Unilateral	A
4	31	Male	C5	C6	R	10	8	Unilateral	A
5	46	Male	C5	C6	R	18	13	Bilateral	A

ASIA describes the level of impairment according to the American Spinal Injury Association classification.

### Equipment

**Figure 1:**
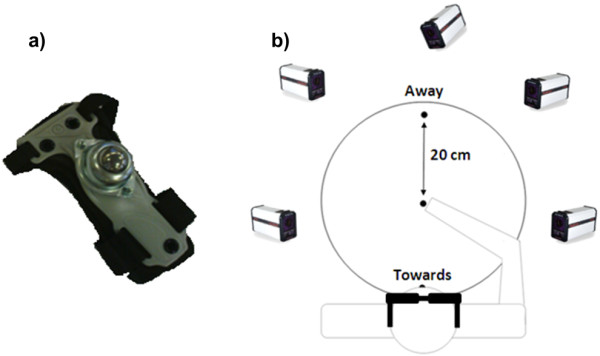
Ball transfer unit with wrist guard (panel a) and target and equipment layout (panel b).

Five Qualisys Pro-Reflex opto-electronic cameras (Qualisys, Gothenburg, Sweden) were used to track the position of a marker on top of a wrist guard at 120 Hz. Attached to the wrist guard was a ball transfer unit (Omnitrack, Woodchester, UK), which consisted of a single large ball bearing partially surrounded by smaller ball bearings (Figure 1a). The line between the marker and large ball intersected the mid-point of wrist. The ball transfer unit allowed participants to perform a ‘frictionless’ multi-directional aiming movement in which they moved their operated (tetraplegics) or dominant (control) arm such that the ball bearing was brought horizontally as fast and as accurately as possible to a target located 200 mm in the sagittal plane, away from or towards the body. This variation of an aiming movement was used because tetraplegic participants had impaired digit control and were unable to perform typical target pointing with the index finger. To indicate the direction and magnitude of movement, a wooden board was placed on top of a table onto which two targets were positioned ±200 mm from a central switch, which acted as the starting position (Figure 1b). The targets and switch were 15 mm diameter, 1 mm thick and required an actuation force equivalent to 200 g (Motion Lab Systems, Baton Rouge, USA). A custom programme in Matlab (v.7.4.0.287 The Mathworks, Inc., Natick, USA) was used to manipulate the availability of visual feedback using Plato liquid crystal glasses (Translucent Technologies, Toronto, Canada) and to specify an auditory start signal.

### Procedure

All participants were seated and the tetraplegic participants remained in their own wheelchair. All participants had trunk movement restricted with a chest strap to avoid trunk motion and thereby facilitate the contribution of elbow motion to task completion. All participants completed a total of 64 horizontal aiming movements, which were separated into four blocks (2 vision, 2 no vision) of 16 trials. Within each block, target location was randomized so that each participant aimed equally away from or towards the body. Blocks were counterbalanced across participants, and advance information was provided regarding availability of vision. Each aiming movement began with the participant pressing down on the central “home” switch with the ball transfer unit, after which there was a random foreperiod between 500–750 ms, followed by an audio cue indicating aiming direction and that the trial should commence. A 400 Hz tone indicated that the participant should aim in the away direction whereas a 200 Hz tone indicated the toward direction. For the trials without vision, the glasses instantaneously became opaque for 5 s once the central “home” switch was released. Participants understood that they were required to perform the aiming task without vision and keep their arm in their final position until the glasses became transparent again. Thus they received terminal visual feedback but not concurrent visual feedback about their aiming. For each combination of condition and aiming direction, participants were required to move the arm as fast and as accurately as possible in order to locate the ball transfer unit on the target. No external feedback was given regarding movement time or final accuracy of the ball-transfer unit relative to the target. Importantly, while the ball transfer unit obscured vision of the target when it was in close proximity, this situation is no different from aiming with the fingertip or a tool to small targets. Also, participants were familiarised with the ball transfer unit and thus the location of the single large ball relative to the wrist-joint centre.

### Data analysis

All data were extracted using Visual 3D (v4.00.20, C-Motion, Germantown, MD, USA) and custom Matlab scripts. The position data from the wrist marker were passed through a Butterworth fourth-order low pass (10 Hz) filter, after which velocity, acceleration and jerk of the wrist marker was calculated using a 2-point finite difference algorithm. A custom-written routine implemented in Matlab then identified movement start and end, which were defined as the moment wrist velocity exceeded, and then fell below, 20 mm/s for 150 ms. Movement time was defined as the interval between movement start and end. Having next identified initial peak in the velocity profile, the routine identified if one of the following events occurred prior to actual end of movement: 1) zero crossing in velocity (i.e., movement reversal); or 2) zero crossing in acceleration; or 3) zero crossing in jerk. The routine searched for these events in the prescribed order, and thus a zero crossing in acceleration or jerk would not be identified if it followed a zero crossing in velocity. If none of the above criteria was met, the aiming attempt was deemed to be completed with a primary movement alone and was not subjected to further analysis. Conversely, if a criterion was met, the trial was deemed to contain a submovement (i.e., movement occurring from the end of the primary movement until movement end), which was classified according to the zero crossing identified; velocity = type 1, acceleration = type 2, jerk = type 3. No search was made for consecutive discontinuities and thus the number of submovements within a trial.

Trials containing a primary movement and submovement were used to calculate a number of dependent variables: percentage of trials with a submovement, percentage distribution of types of submovement (i.e., Type 1, 2 or 3), amplitude of the primary movement (mm) and secondary submovement in the main axis (mm), submovement duration (ms), peak velocity (mm/s), peak acceleration (mm/s^2^), and spatial variability at peak velocity (i.e., standard deviation of the wrist displacement). To test the hypothesis that speed of aiming movement on a trial-by-trial basis may account for the presence of submovements, the relationship between peak velocity of the primary movement and the presence of secondary submovements (yes = 1, no = 0) was examined using a bi-serial correlation for each participant. Finally, to determine if the secondary submovement was functional, we also calculated the number of trials in which: 1) absolute error at the end of the primary movement was greater than absolute error at movement completion (functional); 2) absolute error at the end of the primary movement was less than absolute error at movement completion (non-functional).

Where appropriate, these performance and kinematic variables were analysed in SPSS (v.18, IBM, Armonk, NY, USA) using separate 2 group (tetraplegic, control) x 2 condition (vision, no vision) x 2 direction (away from the body, towards the body) mixed analyses of variance with repeated measures on the last two factors. Post hoc tests were conducted using the Tukey HSD procedure. To ensure normality of distribution, correlation coefficients were converted to Fisher Z scores and percentage data were subjected to arcsine transformation. Alpha was set at *P* < 0.05.

## Results

**Figure 2:**
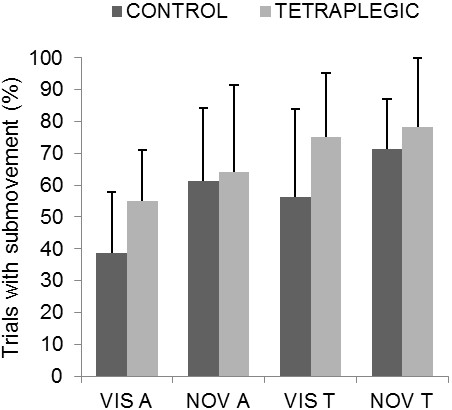
**Percentage of trials that contained a submovement.** Data are presented for both groups as a function of condition and direction (VIS A, Vision Away; NOV A, No Vision Away; VIS T, Vision Towards; NOV T, No Vision Towards). Error bars represent one standard deviation.

As can be seen in Figure 2, the percentage of trials containing a submovement did not differ significantly between the tetraplegic (68%) and control (57%) groups [F(1,8) = 1.18, *P* > 0.1]. However, for both groups there was a greater percentage of submovements when aiming towards (70%) rather than away (55%) from the body [F(1,8) = 18.37, *P* < 0.01], and without (69%) compared to with (56%) vision [F(1,8) = 5.82, *P* < 0.05].

**Figure 3:**
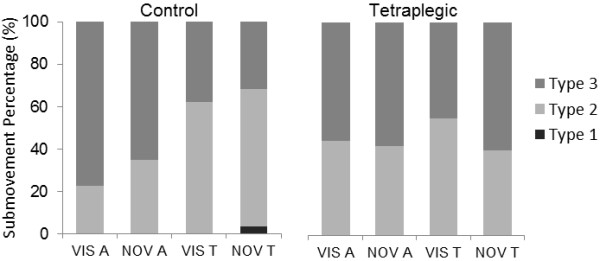
**Percentage distribution of different types of submovements (Type 1 – Velocity; Type 2 – Acceleration; Type 3 – Jerk).** Data are presented for both groups as a function of condition and direction (VIS A, Vision Away; NOV A, No Vision Away; VIS T, Vision Towards; NOV T, No Vision Towards).

Of the trials that contained a submovement, only two from a single subject in the control group were classified as type 1. Therefore, as almost all submovements were manifest in the acceleration (type 2) and jerk (type 3) profiles (Figure 3), we examined if the percentage distribution of the latter differed as a function of the independent variables. For percentage of type 3 submovements, there was a significant main effect of direction [F(1,8) = 5.49, *P* < 0.05], indicating that both groups made more type 3 submovement corrections when aiming away (65%) than toward (47%) the body (Figure 3). There was also a condition × group interaction [F(1,8) = 5.33, *P* <0.05] but post hoc testing did not reveal any significant effects.

Analysis of movement time and peak velocity (Table 2), indicated main effects of group that approached conventional levels of significance [F(1,8) = 4.64, *P* = 0.06 and F(1,8) = 3.99, *P* = 0.08, respectively]. There was a main effect of direction for movement time [away = 645 ms, toward = 600 ms; F(1,8) = 6.12, *P* < 0.05], and a condition × direction × group interaction for both movement time [F(1,8) = 12.76, *P* < 0.01] and peak velocity [F(1,8) = 6.30, *P* < 0.05]. The tetraplegic and control group exhibited longer movement times when aiming away from the body, although for tetraplegics this was particularly evident without vision. A similar pattern of effects were evident in peak velocity. ANOVA on peak acceleration indicated main effects of group and direction [F(1,8) = 8.813, *P* < 0.02 and F(1,8) = 11.142, *P* < 0.02, respectively]. Tetraplegics exhibited lower peak acceleration than controls (3291 mm/s^2^, 8277 mm/s^2^), while peak acceleration of both groups was greater when aiming toward than away from the body (6284 mm/s^2^, 5284 mm/s^2^).

**Table 2:** Mean (±standard deviation) kinematic variables for control and tetraplegic participants as a function of condition and direction (VIS A - Vision Away; NOV A - No Vision Away; VIS T - Vision Towards; NOV T - No Vision Towards)

		**VIS A**	**NOV A**	**VIS T**	**NOV T**
Movement time (ms)	Control	570 (81)	560 (92)	517 (39)	543 (51)
	Tetraplegic	703 (134)	747 (144)	678 (153)	662 (172)
Peak Velocity (mm/s)	Control	895 (197)	841 (250)	921 (205)	924 (253)
	Tetraplegic	646 (178)	589 (171)	587 (176)	602 (199)
Peak Acceleration (mm/s^2^)	Control	8289 (3695)	7881 (4376)	9523 (3137)	9957 (4463)
	Tetraplegic	2987 (833)	3029 (551)	3511 (1604)	3549 (1510)
Primary Movement Amplitude (mm)	Control	191 (9)	192 (5)	176 (9)	183 (12)
	Tetraplegic	190 (15)	178 (15)	157 (30)	162 (22)
Submovement Amplitude (mm)	Control	14 (7)	11 (4)	14 (5)	14 (7)
	Tetraplegic	16 (6)	25 (10)	32 (24)	28 (17)
Submovement Duration (ms)	Control	125 (36)	108 (23)	127 (30)	133 (33)
	Tetraplegic	153 (33)	199 (82)	203 (101)	186 (84)
Spatial Variability at Peak Velocity (mm)	Control	10 (7)	9 (3)	9 (4)	11 (6)
	Tetraplegic	12 (5)	19 (8)	21 (10)	15 (2)

For primary movement amplitude, there was a significant main effect of direction [F(1,8) = 6.07, *P* < 0.05], as well as a condition × direction interaction [F(1,8) = 5.50, *P* < 0.05]. Post-hoc testing showed that primary movement amplitude was greater when aiming away from (188 mm) than toward (169 mm) the body, and that this difference was somewhat larger in the vision than no vision condition (see Table 2). The main effect of group for primary movement amplitude approached conventional significance, with tetraplegics tending to make smaller amplitude primary movements than controls (172 mm vs. 185 mm, F(1,8) = 4.30, *P* = 0.07). Analysis of submovement amplitude revealed a significant main effect of group, with tetraplegics making larger corrections than controls (25 vs. 14 mm; F(1,8) = 6.01, *P* < 0.05). For duration of corrective submovements, the main effect of group also approached significance, with tetraplegics tending to exhibit a longer correction time than controls (185 ms vs. 123 ms; F(1,8) = 3.98, *P* = 0.08). These effects indicate that the tetraplegic group tended to make a smaller amplitude primary movement, which was followed by a larger amplitude and longer duration secondary submovement.

For magnitude of spatial variability at peak velocity in submovement trials there was a significant main effect of group [F(1,8) = 6.232, *P* < 0.05], and significant condition × direction × group interaction [F(1,8) = 6.781, *P* < 0.05]. Overall, the tetraplegic group (17 mm) had greater spatial variability at peak velocity than the control group (10 mm). Also, while spatial variability at peak velocity in the control group was consistent across vision conditions and aiming direction, the tetraplegic group exhibited greatest variability when aiming away from the body with no vision and towards the body with vision (Table 2).

The results of the bi-serial correlation indicated that across each combination of vision and direction, there were no significant within-participants relationships for the tetraplegic group (grand mean Fisher Z score = 0.01) or control group (grand mean Fisher Z score = −0.33). The same pattern of results was found for bi-serial correlations between peak acceleration of the primary movement and the presence of submovements (tetraplegic group: grand mean Fisher Z score = 0.03; control group: grand mean Fisher Z score = −0.26). Therefore, having shown that peak velocity of the primary aiming movement within a trial was not related to the incidence of a submovement, we sought to determine if the submovements were functional in reducing end-point error. To this end, we calculated the percentage of trials in which: 1) absolute error at the end of the primary movement was greater than absolute error at movement completion (functional); 2) absolute error at the end of the primary movement was less than absolute error at movement completion (non-functional). These data were treated as an additional repeated measure (function) in our ANOVA design, which indicated a significant difference in the overall percentage of functional (mean = 69%) vs. non-functional (mean = 31%) submovements [F(1,8) = 15.10, *P* < 0.01]. While there was no effect of group, there was a significant interaction between function, condition and direction [F(1,8) = 5.35, *P* < 0.05]. Post hoc testing indicated that both groups made a greater percentage of functional (mean = 84%) than non-functional (mean =16%) corrections when aiming toward irrespective of vision. The difference between functional and non-functional corrections when aiming toward was larger in the vision than no vision condition but both were significant. There was no difference between functional (mean = 55%) and non-functional (mean = 46%) corrections in the away condition, irrespective of vision condition.

Finally, constant error at movement completion was examined to determine the effectiveness of submovements when present. ANOVA indicated a main effect of direction only [F(1,8) = 12.340, *P* < 0.01], which was reflective of overshoot when aiming away from the body (grand mean = 4 mm) and undershoot when aiming toward the body (grand mean = −8 mm). There was no difference between the groups or any advantage of having vision while aiming.

## Discussion

Goal-directed aiming movements performed by C6 tetraplegics who have undergone tendon transfer surgery (i.e., posterior deltoid replaces the elbow extensor function of the triceps) are of longer duration and lower peak velocity than those performed by age-matched neurotypical controls [5]. Such movement kinematics could reflect a limb control strategy of tetraplegic participants that minimizes the spatial variability associated with a noisier neuromuscular system [8,9,11]. However, slower aiming movement of tetraplegics compared to neurotypicals could simply be a result of adapted motor control and thus could be accompanied by a different distribution of functional and non-functional submovements [11,12]. Here, then, to better understand the control of goal-directed aiming movements in tetraplegics with PD transfer, we report for the first time a detailed analysis of the frequency and type of submovements, as well as the movement kinematics in only those trials containing a submovement phase. These data are compared to those of a neurotypical control group, and related to other measures of motor control in order to better understand the adapted motor control of tetraplegics with PD transfer.

**Figure 4:**
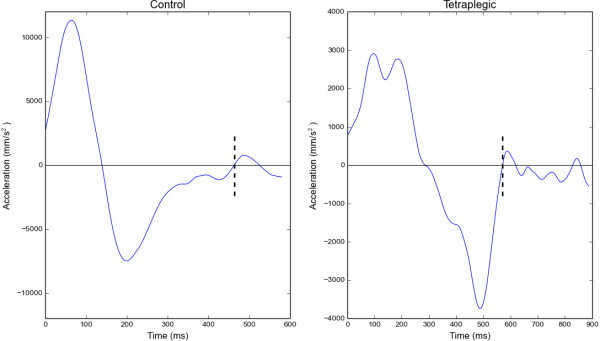
**Representative acceleration profiles for a single participant from the control and tetraplegic groups performing in Vision-Away condition.** Submovement occurrence is indicated by dashed vertical lines.

Consistent with a previous comparison of neurotypical young and older adults performing discrete aiming to a small target [12], we found the tetraplegic (68%) and control (57%) groups exhibited submovement corrections in more than half of the trials. This is somewhat greater than in previous studies [5], where only a coarse identification of submovements were considered (i.e., type 1 and type 2). Importantly, however, we once again found no difference between the groups, and further that there was a greater percentage of submovements when aiming towards (70%) rather than away (55%) from the body. In terms of the distribution, only a single participant in the control group made a type 1 submovement (i.e., zero crossing in velocity). This lack of large overshoot errors followed by type 1 submovements is consistent with work on discrete aiming where the movement is performed in the same horizontal plane as the targets [9,13]. It does, however, contrast with some previous work [14,15] where the horizontal limb movement and targets were presented on a vertically-oriented computer display. In such a situation, it would have been necessary to perform a visuo-spatial transformation, which likely resulted in less optimal visuo-motor control and thus a high prevalence of large overshoot errors followed by movement reversals (i.e., zero crossings in velocity). Also, it should be borne in mind that in the current study participants performed discrete aiming movements with their arm resting on a ball transfer unit, which minimized friction with the aiming surface. These sliding-type movements could have made it easier to keep the arm moving with low velocity around the end of the primary movement and thus facilitated type 2 and 3 submovements.We next examined whether there were differences in the kinematics of submovement trials between the two groups. We found that tetraplegics tended to move with longer duration and reduced peak velocity in submovement trials than control participants. This was more clearly reflected in acceleration, with tetraplegics exhibiting significantly lower peak values than controls. The tetraplegic group also had greater spatial variability at peak velocity than the control group. Spatial variability at peak velocity in the control group was consistent across vision conditions and aiming direction, whereas the tetraplegic group exhibited greatest variability when aiming away with no vision and towards with vision. Analysis of primary movement amplitude revealed a main effect of group that approached significance. Tetraplegics tended to make smaller amplitude primary movements than controls. This was then followed by a significantly larger amplitude submovement, which tended to have longer duration than that of the control group. In combination, these data indicate that although there was no difference between the groups in overall percentage of trials containing submovements, the tetraplegic group were subtly different from the control group in several kinematic measures of aiming behaviour (see Figure 4 for representative acceleration profiles).

We then sought to determine whether trials containing submovements were associated with low movement speed. To this end, we performed individual participant analyses to determine if trials containing a submovement had higher velocity (and acceleration) than those completed with a primary movement alone. For both groups, higher speed and acceleration was not associated with the presence of submovements. We then questioned if submovements brought the hand closer to the target [see 10 for the rationale behind our method]. It was found that both tetraplegic and control groups exhibited a greater percentage of trials in which there was a decrease (functional) compared to increase (non-functional) in end-point error following a submovement. In addition, both groups made a greater percentage of functional than non-functional corrections when aiming toward in the vision and no vision conditions. There was no difference between functional and non-functional corrections in the away condition, irrespective of vision condition. These data indicate that submovements made by both groups were equally effective in the presence of vision and/or proprioception, and particularly when aiming toward the body. The similarity in end-point control between tetraplegic and control groups was confirmed by analysis of constant error at movement completion, which revealed a difference in direction only. Both groups exhibited a small overshoot when aiming away from the body and a small undershoot when aiming toward the body. Together, these data indicate that while the tetraplegic group exhibited initial impulse control (i.e., feedforward) that was more variable, this was compensated by the limb-target control phase (i.e., feedback) to achieve similar end-point accuracy as the control group; for a detailed review of feedforward and feedback processes operating in aiming see [7].

We acknowledge that submovements that bring the hand closer to the target could appear to be functional simply because they were identified soon after peak velocity within a trajectory that contains fluctuations (i.e., type 2 and type 3). However, with the target located at only 200 mm from the home position, it would certainly have been possible for participants to make large overshoot errors that required a type 1 (i.e., movement reversal) correction. Such behaviour was not, or at least very rarely, exhibited by either group in the current study. The virtual absence of type 1 submovements is reflective of a strategy often seen in manual aiming where the whole mass of the limb must move though 2-dimensional [16] or 3-dimensioanl [6] space. Specifically, performers often strategically undershoot the target with their primary movement so that time and energy consuming reversals to the direction of the movement are avoided [7]. In the current study, participants came close to the target with the primary movement when aiming toward the body and then made type 2 or type 3 submovements that moved the hand even closer to the target (i.e., functional submovements). When aiming away from the body, participants made a larger amplitude primary movement, which coincided with fewer trials containing a submovement. It is possible that part of the direction difference reflects the temporal and energy costs associated with a movement reversal. That is, having planned to land closer to the target when aiming away, inherent movement variability resulted in some trials overshooting, which participants then chose not to correct because of the need to move the limb a greater distance, as well as overcoming the inertia associated with a zero velocity at the point of a reversal. This strategy would be consistent with the small but reliable positive constant error for away movements exhibited by participants in both groups, and demonstrates excellent spatial awareness and control of the limb on the ball transfer unit in relation to the target.

As alluded to above, the results of the current study provide some insight into feedforward and feedback motor control following the posterior-deltoid transfer. Specifically, one may have expected that feedforward control would be impaired by the transfer such that tetraplegics are unable to generate appropriately timed and scaled muscle forces, thus producing an initial impulse phase that necessitates significantly more correction during the limb-target control phase. Although we found increased spatial variability in the initial phase of reaching, reduced peak acceleration and a tendency for lower amplitude primary movement, there was no difference between the groups in the proportion of trials containing submovements. Moreover, when a submovement was made during the limb-target control phase, these were mostly functional thus indicating tetraplegic participants were able to use visual and/or proprioceptive feedback to aim at the horizontal targets with similar end-point error as neurotypical controls. We have reported a similar movement strategy in 3-dimensional aiming [17], thus adding support to clinical observations of a benefit for PD transfer in functional reaching tasks. As these results are from a small sample, it is not known how generalizable they are to the wider population of tetraplegics with a posterior-deltoid transfer.

In terms of functional implications, it should first be borne in mind that the inertia of the limb did not have to be independently supported in this experiment, unlike in some earlier studies of reaching [17,18]. In this respect, the constraints of the current study are somewhat similar to those that would be experienced by the tetraplegic participants when performing tasks such as manipulating a computer mouse or motorised wheelchair control. This type of supported limb control is therefore important for a number of daily life activities including communication, education and independent transport. The results of the current study indicate that in such tasks, PD transfer enables C6 tetraplegics to effectively use their altered neurology and adapt the function played by the posterior deltoid prior to surgery. In future studies it will be important to determine the timescale for such adaptation. Additionally, more specific insight into the effect of the posterior-deltoid transfer could be gained from comparison to C7 tetraplegics who would have normal elbow extension function. This group would more closely reflect the functional capabilities of the C6 tetraplegics but without the impairment of elbow extension.

## Conclusions

A detailed analysis of trials containing a primary and submovement phase show that although the tetraplegic group moved slower and exhibited more spatial variability during the initial phase of the movement, they achieved comparable end-point error as control participants. The implication is that tetraplegic participants adopt a subtly different but still effective speed-accuracy relation compared to control participants, indicating that feedforward and feedback motor control was both intact and effective.

## Competing interests

The authors declare that they have no competing interests.

## Authors’ contributions

MR conceived of the study, collected and analysed the data and drafted the manuscript. DE conceived of the study, interpreted data, drafted and critically revised manuscript. SH conceived of the study and critically revised the manuscript. GB conceived of the study and critically revised the manuscript. SB conceived of the study, interpreted data, drafted and critically revised the manuscript. All authors read and approved the final manuscript.
